# Phylogenomics databases for facilitating functional genomics in rice

**DOI:** 10.1186/s12284-015-0060-7

**Published:** 2015-07-30

**Authors:** Ki-Hong Jung, Peijian Cao, Rita Sharma, Rashmi Jain, Pamela C Ronald

**Affiliations:** Graduate School of Biotechnology & Crop Biotech Institute, Kyung Hee University, Yongin, 446-701 Republic of Korea; China Tobacco Gene Research Center, Zhengzhou Tobacco Research Institute, Zhengzhou, 450001 China; School of Life Sciences, Jawaharlal Nehru University, New Delhi, 110067 India; Department of Plant Pathology and the Genome Center, University of California, Davis, California 95616 USA; The Joint Bioenergy Institute, Emeryville, California 95616 USA

## Abstract

The completion of whole genome sequence of rice (*Oryza sativa*) has significantly accelerated functional genomics studies. Prior to the release of the sequence, only a few genes were assigned a function each year. Since sequencing was completed in 2005, the rate has exponentially increased. As of 2014, 1,021 genes have been described and added to the collection at The Overview of functionally characterized Genes in Rice online database (OGRO). Despite this progress, that number is still very low compared with the total number of genes estimated in the rice genome. One limitation to progress is the presence of functional redundancy among members of the same rice gene family, which covers 51.6 % of all non-transposable element-encoding genes. There remain a significant portion or rice genes that are not functionally redundant, as reflected in the recovery of loss-of-function mutants. To more accurately analyze functional redundancy in the rice genome, we have developed a phylogenomics databases for six large gene families in rice, including those for glycosyltransferases, glycoside hydrolases, kinases, transcription factors, transporters, and cytochrome P450 monooxygenases. In this review, we introduce key features and applications of these databases. We expect that they will serve as a very useful guide in the post-genomics era of research.

## Review

### Functional redundancy remains a large obstacle in functional genomics studies

Progress in functional genomics studies can be significantly inhibited by functional redundancy existing within a genome. This redundancy can be identified through analysis of genome sequences and transcriptomic data. The whole-genome rice sequence, completed by the International Rice Genome Sequencing Project (IRGSP) consortium, indicates the presence of up to as many as 3,865 paralogous protein families in rice (IRGSP 2005). These include 21,998 proteins out of 42,653 total non-transposable element (non-TE)-related proteins predicted by the Michigan State University Rice Genome Annotation Project (MSU-RGAP; http://rice.plantbiology.msu.edu/) team (Lin et al. 2008). This suggests that a gene within the rice genome has a 51.6 % possibility of being functionally redundant.

Several plant gene family databases are available for such analyses such as GreenPhyl V4 (http://www.greenphyl.org/cgi-bin/index.cgi) (Rouard et al. 2011), Plant Gene Family Database (Sakai et al. 2013), and SALAD Database (http://salad.dna.affrc.go.jp/salad/en/) (Mihara et al. 2010). These databases provide tools for phylogenetic analysis and have been applied to determine the similarity of assigned functions of gene families. However, the presence of predicted genetic redundancy based on intra-family examinations does not always accurately predict functional redundancy. For example, a genome-wide survey of predicted light-responsive genes in rice and functional analysis of T-DNA insertional mutants revealed that four of the tested family members have defects associated with normal growth or chlorophyll biosynthesis (Jung et al. 2008). These genes are highly expressed at leaf tissue. These results suggest that such highly expressed members of a gene family are good targets for functional investigations.

To further explore this idea (Jung et al. 2015), we analyzed the phylogenetic relationship and expression patterns of members of 79 gene families with known function. Of these 79 gene families, 65 carry at least one member that is highly expressed. We found that the redundancy of these families was limited to two or three members of each family. This study confirmed that phylogenomics analysis integrating gene expression data within a phylogenetic context is an effective strategy to select genes for functional genomics studies.

### Construction of phylogenomics databases for six large gene families in rice

**Fig. 1:**
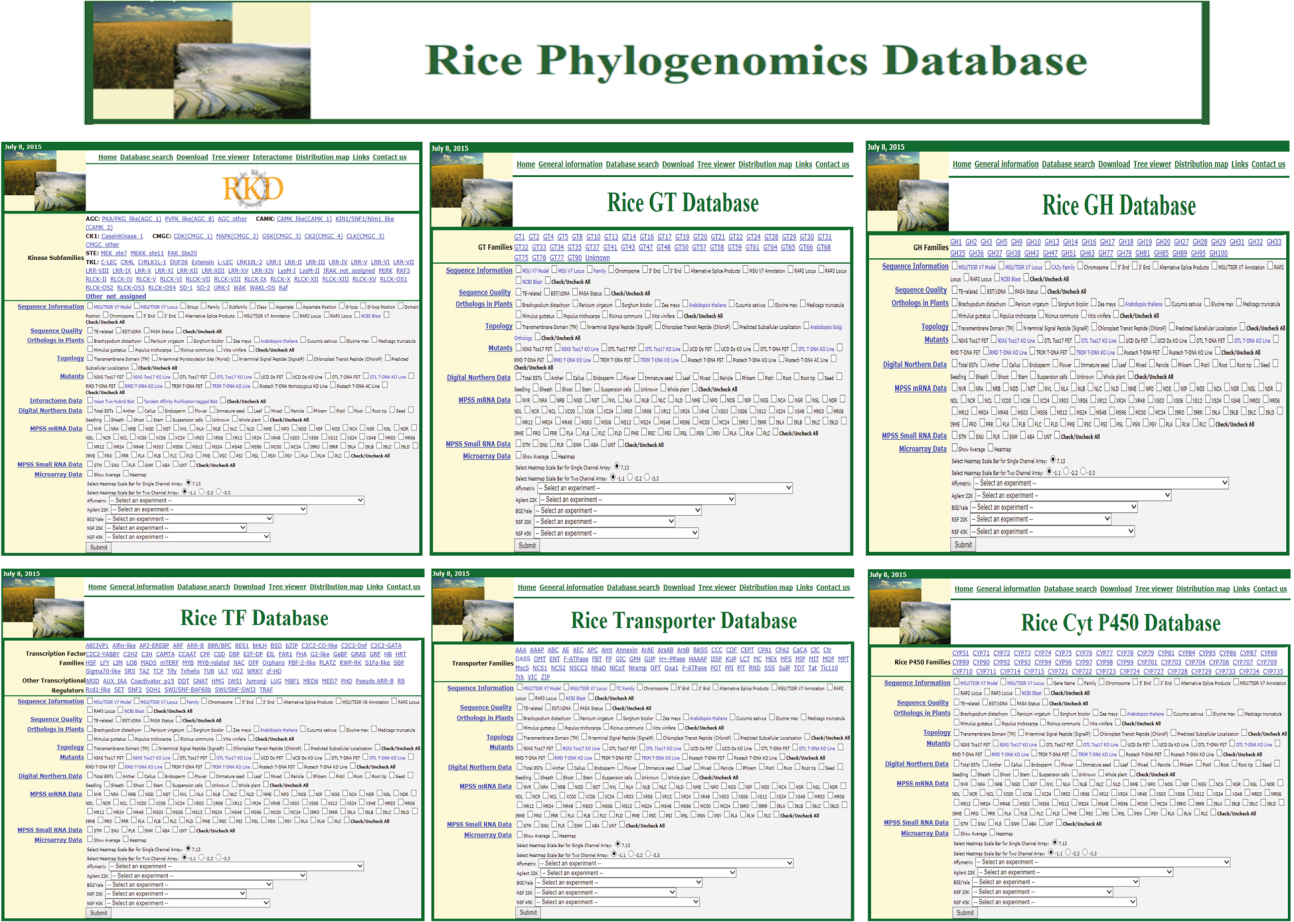
Snapshots from homepage for rice phylogenomics databases and Tree viewer sites for six phylogenomics databases

In rice, phylogenomics databases for six large gene families, i.e., those for kinase, glycosyltransferases (GTs), glycoside hydrolases (GHs), transcription factors (TFs), transporters, and cytochrome p450 monooxygenases (P450s), have been constructed. A description of three of these databases (kinase, GT, and GH) has been reported (Cao et al. 2008; Dardick et al. 2007; Jung et al. 2010; Sharma et al. 2013). Although the other databases have not yet been fully described, some features of those databases are now publicly available. Figure 1 shows the homepage for the rice phylogenomics database s (http://ricephylogenomics.ucdavis.edu/) plus snapshots of Tree viewer webpages for all six databases.

#### Rice Kinase Database

**Fig. 2:**
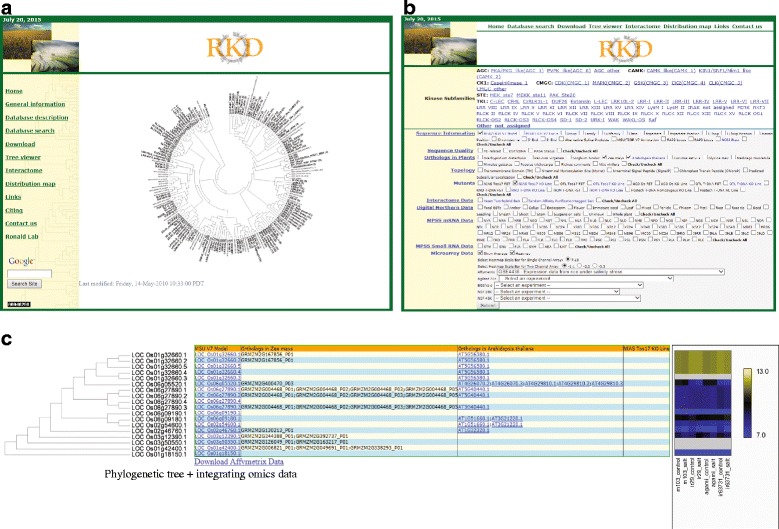
Snapshots from homepage for rice kinase database showing phylogenomics data for cyclin dependent kinase (CDK) subfamily using Tree viewer tool. At Rice kinase database (RKD) homepage (**a**), we clicked Tree viewer option and moved to RKD Tree viewer website (**b**). This website consists of two parts: Kinase subfamilies and integrating data sections. After then, we selected CDK CMGC_1) from kinase subfamilies, MSU/TIGR V7 Model from sequence information, Zea mays and Arabidopsis thaliana from orthologs in plants, transmembrane domain from topology, NIAS Tos17 KO line from mutants, and show average and heatmap of Affymetrix GSE4438 dataseries (**c**). The scale bar for single channel array data from 7 to 13 log2 transformed intensity value was applied. Yellow color indicates the high level of expression and blue one indicates the low level

The Rice Kinase Database (RKD) was created to host various functional genomics data within the context of a phylogenic tree for individual subfamilies. The RKD provides a platform to integrate disparate data sets on such a tree. Those sets include ten types of information (Table 1). Of these, elements that are key to functional genomics studies are the protein–protein interaction data and meta-expression data based on microarray data analyses. The former has 378 interactions revealed by Y2H as well as 254 distinct kinase interactors and 364 interactions via TAP assays. This information is unique to RKD. The latter provides information about 1,867 samples from 105 data series available from the NCBI Gene Expression Omnibus (GEO; http://www.ncbi.nlm.nih.gov/geo/) or the plexDB (http://www.plexdb.org) (Barrett et al. 2011; Dash et al. 2012). Therefore, users can easily estimate the functional redundancy between closely linked family members for the selected kinase. Tree Viewer is the main web tool for integrated analysis; part or all of the listed data for a selected gene family can be seen in a phylogenetic context (Fig. 2). The RKD also includes an interactive chromosomal map showing the positions of all rice kinases and interactive protein–protein interaction maps. Links are provided to navigate around those pages and also to MSU-RGAP. This format simplifies comparisons of closely-related kinases within subfamilies. The RKD has been updated to include rice genome annotations in MSU-RGAP *ver* 7. In that version, 1,467 kinase genes (loci) correspond to 1,934 transcripts (gene models).

**Table 1:** Summary of the types of data integrated in phylogenomics databases

Data type	Providing information or data	Reference
Sequence	locus IDs from MSU-RGAP and The Rice Annotation Project Database (RAP-DB; http://rapdb.dna.affrc.go.jp/), family and sub-family names, domain positions, NCBI blast result	(IRGSP 2005; Yuan et al. 2005)
Sequence Quality	TE-relatedness, existence of EST/cDNA, and Program to Assemble Spliced Alignments (PASA) status	
Orthologs in Plants	orthologs from 12 plant species, (i.e., *Brachypodium distachyon*, *Panicum virgatum*, *Sorghum bicolor*, *Zea mays*, *Arabidopsis thaliana*, *Cucumis sativus*, *Glycine max*, *Medicago truncatula*, *Mimulus guttatus*, *Populus trichocarpa*, *Ricinus communis*, and *Vitis vinifera*)	(Berglund et al. 2008)
Topology	Transmembrane Domain (TM), N-terminal Myristoylation Site (Myrist), N-terminal Signal Peptide (SignalP), Chloroplast Transit Peptide (ChloroP), and predicted Subcellular Localization	
Mutants	mutant lines and corresponding flanking sequence tags from eight institutes	(Chandran and Jung 2014)
Interactome Data	experimentally validated network of protein–protein interactions based on Yeast Two-Hybrid (Y2H) and Tandem Affinity Purification (TAP) methods	(Ding et al. 2009)
Digital Northern Data	normalized frequency of ESTs in selected tissues/organs	(Dardick et al. 2007; Jung et al. 2010)
MPSS mRNA Data	meta-expression data from 70 libraries	(Nakano et al. 2006)
MPSS Small RNA Data	meta-expression data from six libraries	(Nakano et al. 2006)
Microarray Data	meta-expression data from the six microarray platforms including Affymetrix, Agilent22K, Agilent44K, BGI/YALE60K, NSF20K, and NSF45K (http://ricephylogenomics.ucdavis.edu/description.shtml)	(Cao et al. 2012)

#### Rice GT database

Glycosyltransferases constitute a large group of enzymes that form glycosidic bonds through the transfer of sugars from activated donor molecules to acceptor molecules. They are critical to the biosynthesis of plant cell walls. The Rice GT Database was created to integrate and host functional genomics information for all putative rice GTs (Cao et al. 2008). This database contains information about 609 potential GT genes (loci) that correspond to 769 transcripts (gene models). Those loci have been identified from the rice genome through similarity searches that utilized GT sequences available from the Carbohydrate Active enZymes (CAZy) database (http://www.cazy.org/) (Egelund et al. 2004). Based on domain compositions and sequence similarities, we have classified rice GTs into 41 CAZy families, including one unknown class. Following analysis with Inparanoid, we can suggest that 282 'rice-diverged' GTs have no orthologs in sequenced dicot species (e.g., *A. thaliana*, *P. trichocarpa*, *M. truncatula*, and *R. communis*) (Sonnhammer and Ostlund 2014). Similar to the RKD, we have developed a platform to display user-selected functional genomics data on a phylogenetic tree. These include all integrated data except interactome data (http://ricephylogenomics.ucdavis.edu/cellwalls/gt/).

#### Rice GH database

Glycoside hydrolases (GHs) catalyze the hydrolysis of glycosidic bonds in cell wall polymers and, along with GTs, are major contributors to plant cell architecture (Sharma et al. 2013). Several GHs have been identified from the rice genome based on sequence similarity searches that used GH sequences in the CAZy database. The rice genome encodes 437 GH genes corresponding to 614 gene models that have been classified into 34 families. Using the massive datasets available in public databases, we have created a phylogenomics database of rice GHs (http://ricephylogenomics.ucdavis.edu/cellwalls/gh/) that integrates multiple data types. The new sets incorporate structural features, orthologous relationships, mutant availability, and gene expression patterns for each GH family within a phylogenomics context (Sharma et al. 2013). After comparing them with dicot GHs, we believe that 138 GH genes are possibly monocot-diverged. By integrating and analyzing these phylogenetic and expression data, researchers should be able to identify potential targets for engineering cell wall structure and stress tolerance. Other features of the GH database are similar to those of the GT database.

#### Rice TF database

A transcription factor binding to specific DNA sequences controls the rate of transcription of genetic information from DNA to messenger RNA (Todeschini et al. 2014). Rice TFs have been retrieved from the Plant Transcription Factor Database (http://plntfdb.bio.uni-potsdam.de/v3.0/) (Zhang et al. 2011). This Rice TF Database (http://ricephylogenomics.ucdavis.edu/tf/) hosts 2,385 genes corresponding to 3,119 models classified into 80 families. It integrates and provides functional genomics information for all putative rice TFs and other predicted transcriptional regulators. Like other databases, we have integrated multiple data types, such as structural features, orthologous relationships, mutant availability, and gene expression patterns for each TF family within a phylogenomics context. Other features are similar to those of the GT database.

#### Rice transporter database

A transporter is a membrane protein involved in the movement of ions or small molecules (Saier et al. 2014). Transporter proteins exist permanently within and span the membrane across which substances are transferred. The rice genome contains 1,211 potential transporter genes (loci) corresponding to 1754 gene models (Ren et al. 2007). These sequences have been retrieved from the Transporter Protein Analysis Database (TransportDB; http://www.membranetransport.org/), which was created to merge and provide functional genomics information for all putative rice transporters. Like for the other databases, we have integrated multiple data types that include structural features, orthologous relationships, mutant availability, and gene expression patterns for each transporter family (http://ricephylogenomics.ucdavis.edu/transporter/). Other features are similar to those of the GT database.

#### Rice Cytochrome P450 database

Cytochrome P450 monooxygenases belong to the superfamily of proteins containing a heme cofactor. They have roles in the terminal oxidation of electron transfer chains. The rice genome has 302 genes that encode P450s corresponding to 341 transcripts. Rice P450s have been retrieved from the Cytochrome P450 Database (http://drnelson.uthsc.edu/CytochromeP450.html), and mapped onto the MSU-RGAP *ver* 6 genome annotation. The Rice P450 Database (http://ricephylogenomics.ucdavis.edu/p450/) was created to integrate and provide functional genomics information for all putative rice P450s. As with the other databases, we have integrated multiple data types for each P450 family. The other features are similar to those of the GT database.

### Applying a phylogenomics approach to estimate functional redundancy within a gene family

**Fig. 3:**
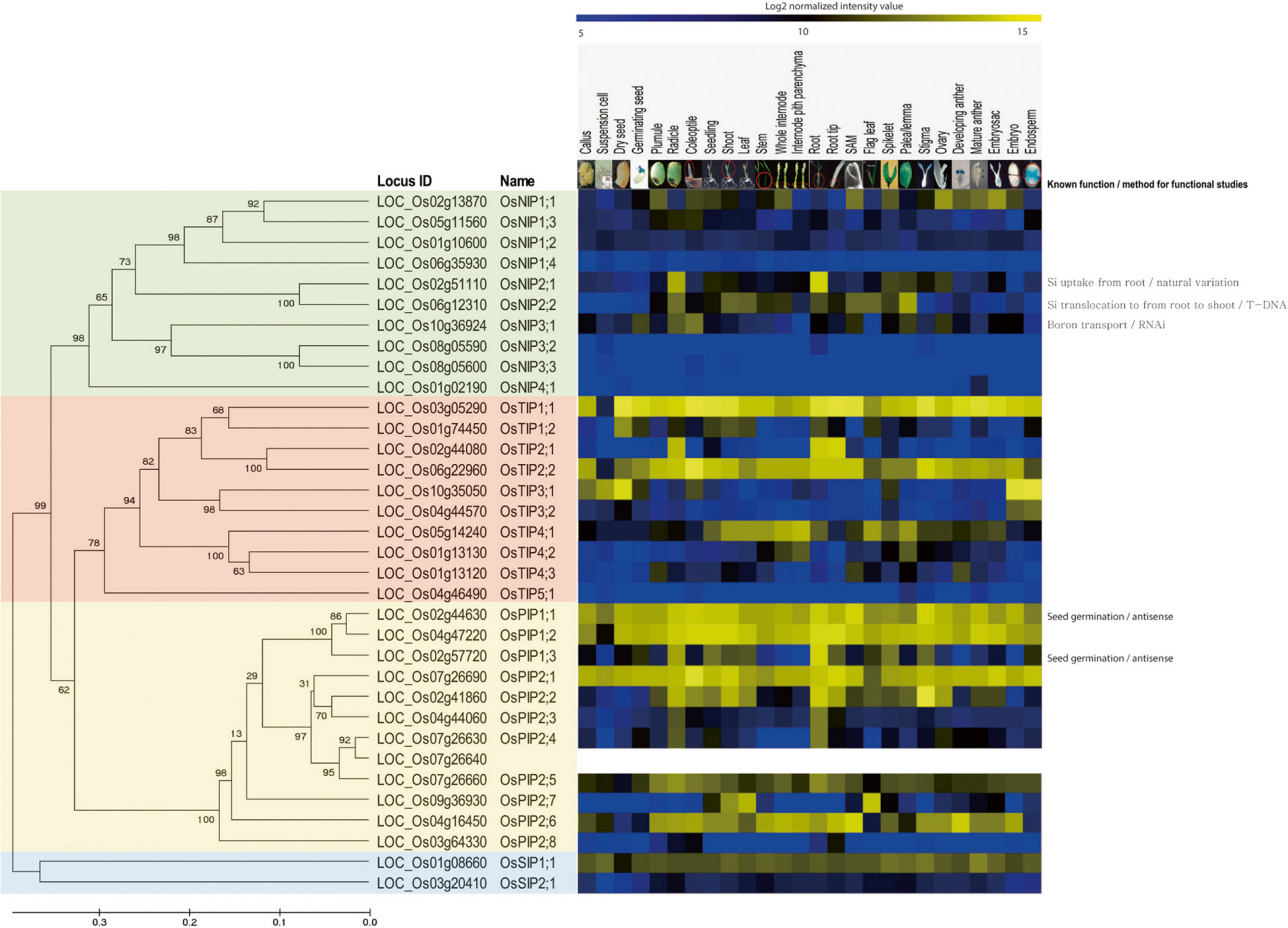
Case study to evaluate the functionality of phylogenomics tool: Phylogenomics analysis of aquaporin family in rice. Anatomical expression data were integrated into phylogenetic tree context of aquaporin family. Anatomical meta-expression data were generated from ROAD with 34 rice aquaporin genes. Their protein classifications revealed 20 NIPs (green boxes), 12 PIPs (pale-yellow boxes), 10 tonoplast intrinsic proteins (pink boxes), and 8 small basic intrinsic proteins (light-blue boxes). For heatmaps, blue color denotes low expression, black medium level expression and yellow indicates high expression. Numbers indicate estimated evolutionary distances

To demonstrate the functionality of the phylogenomics databases, we obtained relevant information, including anatomical meta-expression data in a phylogenetic context, for the aquaporin gene family (Fig. 3) (Nguyen et al. 2013). The functions of five aquaporin gene members have been characterized via loss-of-function studies. Three of the aquaporin gene family members belong to the Nodulin-26 like Intrinsic Protein (NIP) subfamily and two are in the Plasma membrane Intrinsic Protein (PIP) subfamily. One gene, *Low silicon rice1* (*Lsi1*) (*LOC_Os02g51110*), in the NIP subfamily, has been demonstrated to play a role in Si uptake in roots. Anatomical meta-expression data indicate that NIPs show root-preferential patterns of expression that are well-matched with their biological functions. For example, Lsi6 is closely linked to Lsi1 and is involved in Si translocation from roots to shoots. Anatomical meta-expression data reveal that Lsi6 is highly expressed in the root, shoot, leaf, and palea/lemma tissues. Integrated meta-expression data confirms previous reports of distinct functional roles for *Lsi1* and *Lsi6*.

OsNIP3;1 (LOC_Os10g33924) is a boron transporter. P Plant growth cannot be sustained under boron-deficient conditions when expression of that gene is knocked down using RNAi. A phylogenetic tree has indicated that OsNIP3;1, OsNIP3;2, and OsNIP3;3 cluster together. *OsNIP3;1* is the dominantly expressed gene family member (Fig. 3) (Liu et al. 2007). Both *OsPIP1;2* and *OsPIP1;1* are ubiquitously expressed suggesting functional redundancy between the two. Antisense suppression of *OsPIP1;1* causes partially defective phenotypes during seed germination, but overexpression of that gene does not stimulate the occurrence of a more normal phenotype. Even though OsPIP1;3 is closely related to OsPIP1;1 and OsPIP1;2, meta-expression data indicates that *OsPIP1;3* expression is highest in the radical and root. Rice plants that are silenced for *OsPIP1;3* are defective in seed germination whereas overexpression of *OsPIP1;3* enhances germination (Liu et al. 2007). These results demonstrate that *OsPIP1;3* has a major role in seed germination in contrast to the other genes in that clade. These observations confirm that phylogenomic analysis, that integrates global expression data with phylogenetic analysis, is a useful method for identifying distinct roles for closely related gene family members.

### Conclusion and Prospect

Functional genomics studies of rice genes belonging to a single family can be facilitated by phylogenomic analysis that integrates diverse types of biological information in a single page view. Future prospects to advance analysis of gene function include coupling phylogenomic analyses with computational predictions of gene function. For example, we have recently generated a probabilistic functional gene network for rice, called RiceNet (Lee et al., 2011; Lee et al., 2015). We used the GH database phylogenomics database to identify 17 GH glycoside hydrolase gene family members (Sharma et al. 2013). We used these seventeen candidate GH genes to query RiceNet v1. We found that these nine genes are highly predicted to function in the same biological process as cellulose synthase and cellulose synthase-like genes of rice, suggesting a potential role for these nine *GH* genes in cell wall biosynthesis.
